# The Impact of Hyper-Acute Inflammatory Response on Stress Adaptation and Psychological Symptoms of COVID-19 Patients

**DOI:** 10.3390/ijerph19116501

**Published:** 2022-05-26

**Authors:** Ion Papava, Liana Dehelean, Radu Stefan Romosan, Mariana Bondrescu, Cristian Zoltan Dimeny, Eugenia Maria Domuta, Felix Bratosin, Iulia Bogdan, Mirela Loredana Grigoras, Codruta Victoria Tigmeanu, Angelica Gherman, Iosif Marincu

**Affiliations:** 1 Department of Neurosciences-Psychiatry, “Victor Babes” University of Medicine and Pharmacy, Eftimie Murgu Square 2, 300041 Timisoara, Romania; papavaion77@gmail.com (I.P.); lianadeh@umft.ro (L.D.); romosan.radu@gmail.com (R.S.R.); mariana.bondescu@umft.ro (M.B.); 2 Center for Cognitive Research in Neuropsychiatric Pathology, “Victor Babes” University of Medicine and Pharmacy, Eftimie Murgu Square 2, 300041 Timisoara, Romania; 3 Department of Psychiatry, Timis County Emergency Clinical Hospital “Pius Brinzeu”, Liviu Rebreanu 156, 300723 Timisoara, Romania; tzutzuch@yahoo.com; 4 Doctoral School, University of Medicine and Pharmacy “Victor Babes” Timisoara, Eftimie Murgu Square 2, 300041 Timisoara, Romania; 5 Surgery Department, Faculty of Medicine and Pharmacy, University of Oradea, Piata 1 Decembrie 10, 410073 Oradea, Romania; edomuta@uoradea.ro (E.M.D.); imarincu@umft.ro (I.M.); 6 Methodological and Infectious Diseases Research Center, Department of Infectious Diseases, “Victor Babes” University of Medicine and Pharmacy, 300041 Timisoara, Romania; felix.bratosin7@gmail.com (F.B.); iulia-georgiana.bogdan@umft.ro (I.B.); grigoras.mirela@umft.ro (M.L.G.); 7 Department of Anatomy and Embryology, “Victor Babes” University of Medicine and Pharmacy, Eftimie Murgu Square 2, 300041 Timisoara, Romania; 8 Department of Technology of Materials and Devices in Dental Medicine, Multidisciplinary Center for Research, Evaluation, Diagnosis and Therapies in Oral Medicine, “Victor Babes” University of Medicine and Pharmacy, Eftimie Murgu Square 2, 300041 Timisoara, Romania; 9 Research Center for Medical Communication, “Victor Babes” University of Medicine and Pharmacy, Eftimie Murgu Square 2, 300041 Timisoara, Romania; angelaeden30@yahoo.com

**Keywords:** COVID-19, SARS-CoV-2, SCL-90R, COPE-60, severe infection

## Abstract

The severe acute respiratory syndrome coronavirus 2 (SARS-CoV-2) infection induces a significant inflammatory response that are amplified by persistent stress. The pathophysiology of mental illnesses is explored in terms of inflammatory processes. Thus, anxious, depressed, or psychotic episodes may occur as a result of metabolic and immunological imbalances, as a direct result of their effect on the central nervous system, or as a side effect of the COVID-19 medication protocols. As such, the primary objective of this research is to establish if the psychological profiles of COVID-19 patients change substantially according to illness severity. The secondary objective is to determine if particular biological inflammatory indicators are associated with anxiety, sadness, psychoticism, and paranoid ideation. A cross-sectional study was performed on 90 hospitalized patients admitted during a 3-month period in the COVID-19 unit. All patients received the COPE-60 and SCL-90R questionnaires. Clinical and paraclinical data were collected and the information was classified according to the severity of COVID-19.The hyper-acute inflammation encountered in patients with severe COVID-19 infection characterized 80.0% of patients using disengagement coping methods, significantly more than patients with mild or moderate SARS-CoV-2 infection severity (*p*-value = 0.012), respectively, 73.3% severe COVID-19 patients engaging in emotion-focused coping strategies based on the COPE-60 scale (*p*-value = 0.037). Additionally, it was determined that negative coping mechanisms (disengagement) and emotion-focused methods are independent risk factors for developing psychoticism symptoms following acute SARS-CoV-2 infection, based on the SCL-90 questionnaire (OR = 2.07; CI = 1.44–3.01), respectively (OR = 2.92; CI = 1.44–3.01). Elevated white blood cells and monocytes and inflammatory markers, such as fibrinogen, procalcitonin, IL-6, and D-dimers, were also identified as risk factors for psychoticism symptoms in multivariate analysis. It is particularly important to consider the constant mental-state evaluation in patients with severe COVID-19 that might benefit from early intervention before psychotic symptoms onset.

## 1. Introduction

The actual pandemic caused by SARS-CoV-2 virus started in December 2019 with an outburst in Wuhan, China. In February 2020, the new coronavirus disease received the name COVID-19, and the World Health Organization (WHO) announced the possibility of a worldwide expansion of the disease caused by it. After a period of only two months, the pandemic was proclaimed [1]. SARS-CoV-2 infection may include a broad spectrum of manifestations that could be separated into three chronological periods: acute infection, post-acute hyperinflammatory disease, and late-inflammatory and virological sequelae period [2]. During the acute phase, the symptomatology is not very specific, with manifestations ranging from fever, cough, and shortness of breath [3,4] to kidney failure [5] or severe pneumonia, which can evolve into the highly lethal acute respiratory syndrome [6]. Severe symptoms have been found in 5% of people suffering from the disease and in 20% of those admitted to hospital, being in need of intensive care [7]. The overall mortality caused by COVID-19 in September 2020 outreached 1 million worldwide, with significant differences among countries [8]. It appears to have a significant impact on life expectancy; persons dying from it lose on average 11.7 years of life [9].

Apart from its physical impairments, the actual pandemic poses a threat to mental health [10], as a high prevalence of psychiatric symptoms has been noticed in individuals after infection [11], extending from feeling helpless, disturbed sleep patterns, irritability, anger, fear of getting ill or dying to generalized anxiety, depression, suicidal thoughts, post-traumatic stress disorder, and substance abuse [12,13,14]. Moreover, the prolonged restriction measures, isolation, and imposed quarantine [15,16], along with the uncertain course of the disease and the economic crisis, generated tremendous distress [11] in the general population and even more in vulnerable groups. Situations perceived as uncertain or threatening represent the core of chronic stress and anxiety, especially when uncertainty regarding safety is perceived [17]. Thus, when another possibility is not accessible to the brain, an automatic perception of danger is generated, which persists unless inhibited. Although the neurobiological theories argue in favor of default response to stress as a generalized unsafety response [17], psychological theories on resilience suggest that stress response varies according to the strategies developed over time. Therefore, some people use more efficient coping strategies than others when encountering adversity, and several become even more capable of dealing with future stress [18]. Hence, coping may represent personality traits and strategies that people use when facing obstacles or looming events [19]. 

Nonetheless, COVID-19 triggers an important inflammatory response [20] that can be augmented by chronic stress. Inflammatory processes are studied in the pathophysiology of psychiatric diseases [21,22]. Thus, anxious, depressive, and psychotic episodes could also be a result of the metabolic and immunological imbalances, a direct consequence of the central nervous system (CNS), or because of COVID-19 medication [12]. On the other hand, the inflammatory response can be a trigger for mental-health diseases. Based on the aforementioned information, it was hypothesized that inflammation increases the risk of depression, anxiety, and psychosis; thus, patients with hyper-acute inflammation may score higher in SCL-90-R anxiety, depression, psychoticism, and paranoid ideation categories. In other words, the severity of COVID-19 may generate these psychiatric symptoms, making patients vulnerable to future episodes. Additionally, the physical stress caused by the SARS-CoV-2 infection may trigger psychological distress enabling certain coping mechanisms. Therefore, the main end-point of the current study is to determine whether the COVID-19-patients’ profiles differ significantly by disease severity. The secondary aim is to observe the correlation of certain inflammatory markers with anxiety, depression, psychoticism, and paranoid ideation.

## 2. Materials and Methods

### 2.1. Study Design, Setting, and Ethics

A cross-sectional study was performed on COVID-19 patients admitted to “Victor Babes” Infectious Disease and Pneumophtisiology Hospital of Timisoara from 01 April 2021 to 15 June 2021, in collaboration with the Department of Neurosciences-Psychiatry of the Timis County Emergency Clinical Hospital “Pius Brinzeu” in Timisoara, Romania. Both hospitals are affiliated with the “Victor Babes” University of Medicine and Pharmacy in Timisoara, Romania, and operate in accordance with the provisions of Article 167 of Law No. 95/2006, Chapter VIII of Order 904/2006, and the EU Good Clinical Practice Directives 2005/28/EC, the International Conference on Harmonization of Technical Requirements for Pharmaceuticals for Human Use (ICH), and the Declaration of Helsinki—Recommendations for Medical Doctors Conducting Biomedical Research Involving Human Subjects. The Ethics Committee of the “Victor Babes” University of Medicine and Pharmacy in Timisoara, Romania, as well as the Ethics Committees of the affiliated hospitals, accepted the study protocol.

### 2.2. Study Participants, Inclusion Criteria, and Variables

Patients were included in the present study if they tested positive for SARS-CoV-2 infection on at least one Reverse Transcription Polymerase Chain Reaction (RT-PCR) test and had evidence of lung damage on thoracic computed tomography (CT) evaluation comprising ARDS pattern, organizing pneumonia, crazy paving, or other abnormalities. The patients were also evaluated for normal cognitive abilities using the Mini Mental Status Test (MMST) and consented to participate in the study. Patients under the age of 18 years, patients in a critical condition who were unable to comprehend and respond to the survey’s questions, and patients who refused to participate in the research were excluded. Other exclusion criteria comprised invalid or incomplete scales or questionnaires, inadequate patient profiles in terms of imaging examinations and laboratory data that determined the severity of COVID-19, as well as records that lacked patient permission. A convenient sampling method was used to determine the appropriate sample size, with a total of 139 respondents being necessary to fulfill the requirements. At the end of the study, a total of 90 questionnaires were correctly and completely filled for the surveys. 

Clinical and paraclinical data were collected by trained physicians who volunteered to participate in this research. Information was classified according to the severity of the condition as mild, moderate, or severe SARS-CoV-2 infection, as presented in Table 1. At the time of the research, all patients received therapy in accordance with the Romanian Ministry of Health’s COVID-19 standards.

The variables investigated included the following: patient age quantified as a categorical variable (18–39 years old; 40–65 years old; >65 years old); sex (male/female); place of origin (rural/urban); occupation (employed, unemployed, retired, disability retired); comorbidities (cardiovascular and metabolic diseases); serum parameters (white blood cells, fibrinogen, erythrocyte sedimentation rate, c-reactive protein, procalcitonin, platelets, D-dimers, ferritin, interleukin-6); duration of hospital admission; and duration of viral clearance. 

### 2.3. Scales and Questionnaires

The current study relied on the full 60-item survey with 16 scales of the Coping Orientation to Problems Experienced (COPE-60) assessment established by Carver, Scheier, and Weintraub [23]. COPE-60 is a self-reported questionnaire designed to assess the types of coping strategies an individual uses. Thus, it is a qualitative scale, an overall high and low score being irrelevant. A Romanian-validated version of the COPE-60 scale was filled by the participating patients [24]. Each item on the COPE-60 scale may be graded between 1 and 4, where one is equivalent to “I usually don’t do this at all,” and 4 implies “I usually do this a lot.” Coping strategies were classified into several clusters. Firstly, the individual’s response to stress was grouped into engagement vs. disengagement, according to their coping type. The engagement coping cluster included methods, namely, positive reinterpretation and development, emotion concentration and venting, instrumental social support, active coping, restraint, religious coping, humor, emotional and social support, acceptance, suppression of competing activities, and planning. 

By contrast, the coping disengagement cluster referred to the strategies employed to avoid stress and the accompanying emotions, including mental disengagement, behavioral disengagement, denial, and drug use. When one engages or disengages with stress, respondents may deal with it directly (problem-focused coping) or indirectly via the stressor’s related emotions—the discomfort (emotion-focused coping). The emotion-focused cluster of techniques comprises the following: emotional attention and venting, instrumental and emotional social support, denial, and substance abuse. Positive reinterpretation and development, religious coping, humor, suppression of competing activities, and planning were all included in the group of problem-focused coping approaches.

Derogatis created the Symptoms Checklist-Revised (SCL-90-R), which was used in the current study [25]. The Romanian-translated version of the SCL-90-R scale was applied in the present research to ascertain the degree of mental distress by analyzing the kind and intensity of symptoms. This auto-evaluation, multidimensional scale, comprising 90 items, assessed nine major aspects of symptoms in addition to three global pathology indexes. Each item was scored on a 5-point scale ranging from “none at all” (0) to “excessive” (4). Somatization, obsession-compulsion, interpersonal sensitivity, sadness, anxiety, hostility, anxiety phobia, paranoid ideation, and psychoticism were the primary symptomatic groupings. As the aim of the present was to evaluate the extent to which inflammation can be associated with certain psychiatric symptoms observed previously in COVID-19 infection, only four categories of SCL-90-R were used: anxiety, depression, psychoticism, and paranoid ideation. 

SCL-90-R has been verified and certified for use in Romania, with an overall dependability score of more than 0.95 for both Cronbach’s alpha and Guttman’s split-half. Additionally, each of the fourteen measures had high internal validity and integrity, scoring much higher than the 0.7 minimum [26]. A sum of scores of each item was calculated and only scores of 2 or higher were considered as clinically evident symptomatology and reported in tables. 

### 2.4. Statistical Analysis

The statistical analysis was performed using IBM SPSS v.26 (IBM Corp, Armonk, NY, USA). The absolute and relative frequencies of categorical variables were calculated. The chi-squared and Fisher’s tests to compare proportions and the Kruskal–Wallis test was used to compare group differences in nonparametric data. The ANOVA test was used to determine the mean and standard deviation of Gaussian data. The variables determined to be significantly different between comparison groups were included in a multivariate analysis adjusted for confounding factors, with results expressed as odds ratio (OR) and confidence interval (CI).

## 3. Results

A total of 90 patients were surveyed in equal proportions of mild, moderate, and severe cases of COVID-19. The background analysis of patients with SARS-CoV-2 infection presented in Table 2 determined no significant difference between the study groups by age, gender, and place of origin. However, patient occupation, comorbidities, duration of hospital stay, and viral clearance were statistically significantly different between patients with mild, moderate, and severe SARS-CoV-2 infection. A total of 73.3% of patients with mild infection were employed, compared to only 30.0% in the severe infection group, where the majority were retired (36.7%) and on disability retirement (10.0%) (*p*-value = 0.043). Cardiovascular disease was the most frequently found comorbidity among study participants, in a proportion of 63.3% among those who developed a severe SARS-CoV-2 infection, compared to 20.0% in those with mild disease (*p*-value = 0.002). Metabolic diseases were also a significant finding in patients with severe infection, and the proportions between groups were statistically significantly higher in those with moderate and severe infections (*p*-value = 0.016). Hospitalization and viral clearance were significantly longer in patients with severe COVID-19 when compared to cases of mild and moderate diseases (18 days vs. 14 days vs. 10 days, *p*-value < 0.001), respectively (14 days, vs. 12 days, vs. 9 days, *p*-value < 0.001).

A comparison of biological parameters presented in Table 3 identified significant variations outside the normal range between the study groups comprising mild, moderate, and severe COVID-19 cases. Patients in the severe COVID-19 group had a significantly higher count of monocytes and white blood cells, compared to those presenting with a mild or moderate form of the disease (73.3% cases outside normality, *p*-value = 0.049), respectively (86.7% cases outside normality, *p*-value = 0.018). The inflammatory markers fibrinogen, CRP, procalcitonin, and IL-6 were also significantly elevated outside the normal range in patients with severe SARS-CoV-2 infection, compared to the other study groups. Another significant finding was the number of patients in the severe group with elevated D-dimers, compared to the mild and moderate cases (33.3% vs. 13.3%, *p*-value = 0.003).

A comparison of the psychological symptoms and coping mechanisms by the COPE-60 scale, and, respectively, SCL-90 scale determined that 80.0% of patients with hyper-acute inflammatory response during SARS-CoV-2 infection responded to stress with disengagement methods (Table 4). On the contrary, 56.7% of patients with mild COVID-19 rarely used disengagement strategies to cope with stress (*p*-value = 0.012). A total of 73.3% of patients with severe forms of infection often used emotion-focused coping methods, compared to only 46.7% of those with mild disease (*p*-value = 0.037). Additionally, 73.3% of patients with severe COVID-19 developed paranoid ideation, and 26.7% of them developed psychoticism, compared to much lower proportions in the other study groups (*p*-value = 0.017, respectively *p*-value = 0.011).

Table 5 describes the comparison of laboratory parameters stratified by psychological symptoms on the SCL-90 questionnaire. It was observed that patients with psychoticism had a significantly elevated level of monocytes and white blood cells (median = 2.7 × 10^3^/mm^3^, IQR = [1.1–4.8]), respectively (median = 14.9 × 10^3^/mm^3^, IQR = [8.9–15.6]), as well as the inflammatory biological parameters ESR (*p*-value = 0.044), D-dimers (*p*-value = 0.029), and IL-6 (*p*-value < 0.001).

The same comparison of biological parameters presented in Table 6 was stratified by COPE-60 results into four categories of coping strategies to stress. Patients using disengagement as a coping strategy had significantly higher monocyte and white blood cell counts (median = 2.9 × 10^3^/mm^3^, IQR = [1.0–4.9]), respectively (median = 14.9 × 10^3^/mm^3^, IQR = [7.7–19.8]). The median values of inflammatory markers procalcitonin (*p*-value < 0.001) and IL-6 (*p*-value < 0.001) were also found to be significantly different between the four categories of coping mechanisms determined by the COPE-60 questionnaire.

A risk factor analysis for developing psychoticism during SARS-CoV-2 infection was conducted in a univariate and multivariate fashion. The multivariate analysis by COPE-60 results determined that the disengagement (OR = 2.07; CI = 1.44–3.01) and emotion-focused (OR = 2.92; CI = 1.44–3.01) coping mechanisms are independent risk factors for developing psychoticism. From the group of biological parameters, white blood cells and monocytes were identified as independent risk factors for developing psychoticism in SARS-CoV-2 infected patients (OR = 3.42; CI = 2.07–4.62), respectively (OR = 4.38; CI = 3.01–5.64). The inflammatory markers fibrinogen (OR = 1.89; CI = 1.16–3.07), procalcitonin (OR = 2.32; CI = 1.29–3.88), D-dimers (OR = 3.14; CI = 2.06–4.38), and IL-6 (OR = 3.49; CI = 2.28–4.70) also showed a higher likelihood of developing psychoticism during COVID-19 (Table 7).

## 4. Discussion

The current study managed to demonstrate how the hyper-acute inflammation caused by SARS-CoV-2 infection that characterizes patients in the severe COVID-19 group creates a higher likelihood of using negative coping mechanisms. Moreover, the negative coping mechanisms (disengagement) and emotion-focused methods were identified as independent risk factors for developing psychoticism. Although this finding does not prove causality, it suggests the important role of a severe inflammatory status on the way patients deal with a certain level of stress and how it determines the patient mental status. Another important finding of this research is the association of several biological parameters with psychological symptoms and coping mechanisms for stress. The elevated white blood cell count, especially monocytes, was significantly higher in patients with paranoid ideation and psychoticism, as well as being more elevated in severe COVID-19 patients.

Even the differences did not reach statistical significance; in the present research, hyper-acute inflammatory response to COVID-19 were more frequently encountered in the male gender (63.3%) in comparison to women, increasing the mortality risk in this group. Additionally, a study conducted on the Romanian population found that the male gender was a major mortality factor in COVID-19 infection [27]. In the current study, the severe COVID-19 group dominantly comprised retired (36.7%) and disability retired subjects (10.0%), while 73.3% of the subjects with mild forms of infection were employed. Likewise, unemployed and retired subjects were found to be affected by more severe forms of COVID-19 infection, which may also be explained by the frequent presence of chronic comorbidities in these groups [28]. With respect to comorbidities, the most frequently encountered in the current study was cardiovascular disease, observed in 63.3% of those with severe SARS-CoV-2 infection. Following this was the metabolic disease, significantly found more often in subjects with moderate and severe forms of infection in comparison with those from the mild group. Similar to the present research, hypertension and diabetes were directly involved in the mortality risk of patients with SARS-CoV-2 infection [27]. Furthermore, a systematic review and meta-analysis comprising 16 studies and 4448 patients showed an important association between cardiovascular disease and both the severity of the SARS-CoV-2 infection and the increased mortality [29,30]. On the other hand, the most frequent metabolic comorbid states found in the studied sample were diabetes, metabolic syndrome, and thyroid dysfunction, contrary to other studies in which fatty liver disease and hepatitis C were additional risk factors for severity and mortality in COVID-19 infection [31,32,33]. Not least, hospitalization and viral clearance were significantly longer in patients with severe COVID-19 when compared to cases of mild and moderate forms.

The ongoing pandemic raised immense concerns regarding mental health, creating the background for the development of psychiatric illnesses. Consequently, people without any diagnosed condition became susceptible to anxiety and depression, those suffering from chronic somatic diseases had increased feelings of sadness and suicidal ideation, while the ones with severe psychiatric diseases had frequent states of agitation, anxiety, and auto-aggressive behavior [34]. Although the actual coronavirus outbreak affected hundreds of countries, infecting 497 million people worldwide [35], with different disease severity and distinct outcomes, few studies focused on the relationship between patients’ coping strategies for COVID-19 disease severity.

Coping is usually defined by the cognitive and behavioral strategies someone uses to overcome or diminish difficult or stressful events. The strategies may be active or passive, dealing with either the adversity itself or with its repercussions. Even though the stability of coping mechanisms is still under debate, some of them are considered to be adaptive, positively reducing the impact of the distress, while others are considered to be maladaptive, perpetuating the suffering [36]. In the present study, subjects dealing with mild forms of infection used less disengagement, more engagement, and problem-focused strategies, those suffering from moderate forms tended to use engagement and disengagement in similar percentages, while those with severe SARS-CoV-2 infection used more disengagement and emotion-focused coping strategies. A previous study on acute and remitted patients, with similar results, found that remitted patients who suffered from severe forms of SARS-CoV-2 infection used disengagement and emotion-focused coping mechanisms more often [37]. Another study using the Stress and Coping Inventory on the general population during the lockdown found alcohol and cigarette consumption (substance use) as being a predictor of a poor psychological state, depression, and anxiety. On the other hand, social support coping was beneficial for a good mental state [38,39]. Not least, the support for faith coping strategy (religious coping) has been associated with high perceived stress, anxiety, and depression [40]. Previous studies performed on non-infected subjects found higher levels of anxiety and depression in women in comparison to men [39,41]. Positive reframing, acceptance, and humor were associated with less psychological distress, while self-blame, venting, behavioral disengagement, and self-distraction were associated with decreased mental health [41]. Moreover, non-infected subjects with significant psychological distress are more likely to use disengagement (denying problems, behavioral disengagement, substance use) and emotion-focused coping styles (emotional discharge and self-blame) [39]. A study performed on the general population evaluated the way that worries arbitrate the relationship between coping and anxiety, showing that worry augmented the negative effect of dysfunctional coping on anxiety. Contrary, people’s anxiety seems to be increased by problem-focused and decreased by emotional-focused coping styles [42].

Already overstudied, the role of inflammation in psychiatric diseases had not been clearly established. Concerning psychosis, several hypotheses have been drawn about intricate mechanisms that interact, resulting in clinical symptoms of hallucination, delusions, disorganized thought processes, and behavior. Certain inflammatory molecules, such as cytokines, monocytes, macrophages, and lymphocytes, are known to be capable of causing inflammation, including in the Central Nervous System (CNS). However, inflammation is a fundamental mechanism in adaptation; when uncontrolled, it may generate significant damage. The present findings have raised a warning regarding psychotic symptoms in patients with severe forms of SARS-CoV-2 infection, as white blood cells and monocytes were identified as independent risk factors for the development of psychotic symptoms. Other studies have shown the presence of pro-inflammatory markers in the blood and cerebrospinal fluid of patients with schizophrenia [21,43]. In addition, in the present study, increased levels of fibrinogen, procalcitonin, D-dimers, and IL-6 in patients with SARS-CoV-2 infection have also shown a higher likelihood of developing psychotic symptoms. Another study on patients with COVID-19 found structured delusions mixed with confusional symptoms as prevalent psychiatric signs. These could be caused by the presence of the virus in the CNS or because of the viral medication [44]. On the other hand, it is well-known that infection and stress may be triggers for psychotic episodes, especially in vulnerable persons. A systematic review and meta-analysis of 16 included studies that reported elevated IL-6 levels in subjects at clinical risk for psychosis [45]. Considering that previous studies noted increased IL-6 levels in patients suffering from depression [46], this also could be explained by the co-occurrence of depressive and psychotic symptoms in patients with SARS-CoV-2 infections. Not least, previous short psychotic and depressive episodes create the background for chronic psychiatric diseases, such as schizophrenia and major depression.

The limitations of this study warrant a mention. Firstly, the relatively small number of subjects, especially after they were assigned into groups of disease severity, determines an increased risk for type two errors and dims the statistical power of the study. The subjectivity of SCL-90R, as it is an auto-evaluation instrument that increases the bias risk. Lastly, the cross-sectional design can be regarded as a limiting factor as it does not permit the evaluation of the coping and the psychiatric symptoms in a dynamic manner.

## 5. Conclusions

In short, there are several characteristics that may increase the risk of developing hyperacute inflammatory responses to COVID 19, such as a vulnerable status and a dysfunctional appraisal. Thus, more severe forms of infection are found in males, retired and disability retired subjects, as well as in those suffering from cardiac and metabolic diseases. In other words, unemployment/retirement and somatic comorbidities, such as cardiac and metabolic, increase the risk for severe SARS-CoV-2 infection. On the other hand, subjects with hyper-acute inflammatory response are prone to use dysfunctional coping strategies. Thereby, disengagement and emotion-focused strategies put them at risk for psychotic episodes. Not least, increased levels of white blood cells, especially monocytes, were found in patients with paranoid ideation and psychoticism, reinforcing the role of inflammation in psychiatric diseases. Further research on larger sample sizes, including more covariates, can be supportive of validating the present findings.

## Figures and Tables

**Table 1:** COVID-19-severity categories.

COVID-19	Characteristics
Mild	Absence of imagistic symptoms Absence of pulmonary lesions Pneumonia affecting less than 20% of the lung area SpO_2_ of more than 94 percent Absence or mild inflammatory response
Moderate	Impacted lung area between 20% and 50% SpO_2_ between 94% and 87% The need for oxygen treatment for a brief duration Enhanced inflammatory syndrome
Severe	Hyper-acute inflammatory response Affected lung area >50% Required prolonged oxygen treatment

**Table 2:** Background data of study participants by the severity of COVID-19.

Variables *	Mild (*n* = 30)	Moderate (*n* = 30)	Severe (*n* = 30)	*p*-Value **
**Age, years**				0.126
18–40	13 (43.3%)	7 (23.3%)	4 (13.3%)	
40–65	9 (30.0%)	12 (40.0%)	14 (46.7%)	
>65	8 (26.7%)	11 (36.7%)	12 (40.0%)	
**Sex**				0.725
Male	16 (53.3%)	18 (60.0%)	19 (63.3%)	
Female	14 (46.7%)	12 (40.0%)	11 (36.7%)	
**Place of origin**				0.529
Rural	7 (23.3%)	11 (36.7%)	9 (30.0%)	
Urban	23 (76.7%)	19 (63.3%)	21 (70.0%)	
**Occupation**				**0.043**
Employed	22 (73.3%)	12 (40.0%)	9 (30.0%)	
Unemployed	4 (13.3%)	7 (23.3%)	7 (23.3%)	
Retired	3 (10.0%)	9 (30.0%)	11 (36.7%)	
Disability retirement	1 (3.3%)	2 (6.67%)	3 (10.0%)	
Comorbidities ***				
Cardiovascular	6 (20.0%)	11 (36.7%)	19 (63.3%)	**0.002**
Metabolic	5 (16.7%)	8 (26.7%)	15 (50.0%)	**0.016**
**Hospital stay, days (mean ± SD)**	10 ± 3.1	14 ± 3.8	18 ± 6.0	**<0.001**
**Viral clearance, days (mean ± SD)**	9 ± 3.0	12 ± 4.2	14 ± 4.8	**<0.001**

* Data reported as *n*(%) unless specified differently; ** Chi-squared test and Fisher’s exact; *** Cardiovascular comorbidities: high blood pressure, chronic heart failure, ischemic heart disease, and stroke; metabolic comorbidities: diabetes mellitus, metabolic syndrome, and thyroid dysfunction.

**Table 3:** Laboratory profile of study participants by the severity of COVID-19.

Variables *	Normal Range	Mild (*n* = 30)	Moderate (*n* = 30)	Severe (*n* = 30)	*p*-Value **
Monocyte (thousands/mm^3^)	0.1–1.0	13 (43.3%)	15 (50.0%)	22 (73.3%)	**0.049**
WBC (thousands/mm^3^)	4.5–11.0	16 (53.3%)	21 (70.0%)	26 (86.7%)	**0.018**
Fibrinogen (g/L)	2–4 g/L	12 (40.0%)	17 (56.7%)	23 (76.7%)	**0.015**
ESR (mm/h)	0–22 mm/hr	14 (46.7%)	16 (53.3%)	21 (70.0%)	0.171
CRP (mg/L)	0–10 mg/L	9 (30.0%)	15 (50.0%)	19 (63.3%)	**0.033**
Procalcitonin (μg/L)	0–0.5 μg/L	4 (13.3%)	7 (23.3%)	14 (46.7%)	**0.012**
Platelets (thousands/mm^3^)	150–450	3 (10.0%)	8 (26.7%)	12 (40.0%)	**0.028**
D-dimers (ng/mL)	<250	1 (3.3%)	3 (10.0%)	10 (33.3%)	**0.003**
Ferritin (ng/mL)	20–250	3 (10.0%)	4 (13.3%)	6 (20.0%)	0.532
IL-6 (pg/mL)	0–16 pg/mL	5 (16.7%)	8 (26.7%)	14 (46.7%)	**0.035**

* Data reported as *n* (% outside normality) unless specified differently; ** Chi-squared test and Fisher’s exact; WBC—White Blood Cells; ESR—Erythrocyte Sedimentation Rate; IL-6—Interleukin 6.

**Table 4:** Psychometric scale results of study participants by the severity of COVID-19.

Variables		Mild (*n* = 30)	Moderate (*n* = 30)	Severe (*n* = 30)	*p*-Value
**COPE-60**					
Disengagement (2)					**0.012**
	>Median	13 (43.3%)	18 (60.0%)	24 (80.0%)	
	≤Median	17 (56.7%)	12 (40.0%)	6 (20.0%)	
Engagement (3)					0.573
	>Median	18 (60.0%)	20 (66.7%)	16 (53.3%)	
	≤Median	12 (40.0%)	10 (33.3%)	14 (46.7%)	
Emotion Focused (2)					**0.037**
	>Median	14 (46.7%)	13 (43.3%)	22 (73.3%)	
	≤Median	16 (53.3%)	17 (56.7%)	8 (26.7%)	
Problem Focused (3)					0.429
	>Median	17 (56.7%)	14 (46.7%)	12 (40.0%)	
	≤Median	13 (43.3%)	16 (53.3%)	18 (60.0%)	
**SCL-90, median [IQR]**		1.77 [1.09–2.96]	2.25 [1.21–3.18]	2.94 [1.72–3.96]	**<0.001**
Depression		4 (13.3%)	7 (23.3%)	11 (36.7%)	0.107
Anxiety		15 (50.0%)	19 (63.3%)	20 (66.7%)	0.378
Paranoid ideation		12 (40.0%)	13 (43.3%)	22 (73.3%)	**0.017**
Psychoticism		1 (3.3%)	2 (6.7%)	8 (26.7%)	**0.011**

COPE—Coping Orientation to Problems Experienced Inventory; SCL-90—Symptom Checklist-90.

**Table 5:** Comparison of laboratory parameters stratified by psychological symptoms on the SCL-90 scale.

Variables *	Normal Range	Depression	Anxiety	Paranoid Ideation	Psychoticism	*p*-Value
Monocyte	0.1–1.0 thousands/mm^3^	0.8 [0.3–1.2]	0.9 [0.2–1.4]	1.8 [0.6–3.2]	2.7 [1.1–4.8]	**<0.001**
WBC	4.5–11.0 thousands/mm^3^	11.6 [7.3–13.9]	11.4 [7.0–12.2]	13.6 [8.1–14.7]	14.9 [8.9–15.6]	**0.026**
Fibrinogen	2–4 g/L	2.2 [0.5–4.1]	3.0 [0.7–5.2]	4.4 [1.3–6.0]	4.9 [1.7–7.5]	0.138
ESR	0–22 mm/hr	24.8 [16.3–31.5]	25.2 [15.4–33.8]	22.7 [13.1–29.6]	29.4 [18.0–39.7]	**0.044**
CRP	0–10 mg/L	22.0 [11.8–34.3]	26.8 [13.2–37.2]	23.9 [13.6–33.1]	29.1 [15.8–40.3]	0.185
Procalcitonin	0–0.5 μg/L	14.3 [8.1–22.6]	12.7 [7.4–24.9]	16.4 [8.8–27.2]	18.3 [8.2–31.4]	0.063
Platelets	150–450	263 [199–372]	259 [184–337]	246 [180–325]	239 [172–321]	0.692
D-dimers	<250 ng/mL	289 [164–493]	346 [201–528]	302 [196–418]	294 [171–397]	**0.029**
Ferritin	20–250 ng/mL	79 [37–155]	84 [49–182]	103 [57–207]	88 [51–194]	0.107
IL-6	0–16 pg/mL	39.4 [17.0–66.7]	42.7 [21.8–80.4]	55.4 [20.9–83.6]	93.3 [42.4–126.0]	**<0.001**

* Kruskal–Wallis test—significance at 0.05; SCL-90—Symptom Checklist-90; WBC—White Blood Cells; ESR—Erythrocyte Sedimentation Rate; IL-6—Interleukin.

**Table 6:** Comparison of laboratory parameters stratified by COPE-60 categories.

Variables *	Normal Range	Disengagement	Engagement	Emotion Focused	Problem Focused	*p*-Value
Monocyte	0.1–1.0 thousands/mm^3^	2.9 [1.0–4.9]	0.8 [0.3–1.1]	1.6 [0.8–2.5]	1.2 [0.5–1.8]	**<0.001**
WBC	4.5–11.0 thousands/mm^3^	14.9 [7.7–19.8]	11.0 [7.2–14.2]	11.6 [7.2–14.7]	15.1 [8.9–15.6]	**0.002**
Fibrinogen	2–4 g/L	2.2 [0.5–4.1]	3.3 [0.8–4.1]	4.1 [1.3–5.8]	4.7 [1.8–7.3]	0.266
ESR	0–22 mm/hr	23.6 [16.3–31.5]	24.1 [15.0–32.4]	22.3 [12.9–29.6]	25.9 [16.6–33.5]	0.184
CRP	0–10 mg/L	21.9 [11.8–34.3]	25.7 [13.9–37.6]	23.9 [13.6–33.1]	29.1 [15.8–40.3]	0.091
Procalcitonin	0–0.5 μg/L	18.5 [8.1–22.6]	12.1 [7.0–23.8]	15.7 [8.2–26.4]	17.4 [8.0–30.9]	**<0.001**
Platelets	150–450	280 [199–372]	244 [176–323]	219 [176–369]	222 [162–301]	0.549
D-dimers	<250 ng/mL	317 [164–493]	330 [216–498]	310 [183–403]	289 [174–386]	0.247
**Ferritin**	20–250 ng/mL	94 [37–155]	88 [52–191]	107 [61–216]	83 [52–188]	0.315
IL-6	0–16 pg/mL	41.6 [17.0–66.7]	43.2 [21.0–81.5]	53.8 [21.6–82.9]	94.7 [41.8–122.8]	**<0.001**

* Kruskal–Wallis test—significance at 0.05; COPE—Coping Orientation to Problems Experienced Inventory; WBC—White Blood Cells; ESR—Erythrocyte Sedimentation Rate; IL-6—Interleukin 6.

**Table 7:** Risk factor analysis for developing psychoticism during SARS-CoV-2 infection.

	Univariate OR (95% CI)	*p*-Value	Multivariate OR (95% CI)	*p*-Value
**COPE-60**				
Disengagement	2.64 (1.82–3.97)	0.004	2.07 (1.44–3.01)	**0.018**
Engagement	2.09 (1.16–3.44)	0.181	1.85 (1.06–2.74)	0.053
Emotion Focused	3.35 (2.24–5.83)	0.001	2.92 (1.26–4.05)	**<0.001**
Problem Focused	1.88 (1.13–6.08)	0.127	1.34 (1.09–2.71)	0.094
**Laboratory**				
Monocyte	4.92 (2.58–6.67)	<0.001	4.38 (3.01–5.64)	**<0.001**
WBC	5.19 (2.26–6.08)	<0.001	3.42 (2.07–4.62)	**<0.001**
Fibrinogen	3.07 (2.19–4.86)	0.002	1.89 (1.16–3.07)	**0.036**
ESR	1.64 (1.73–2.44)	0.009	1.09 (0.92–1.36)	0.268
CRP	1.79 (1.21–2.50)	0.002	1.26 (0.88–1.54)	0.107
Procalcitonin	3.03 (2.07–4.96)	<0.001	2.32 (1.29–3.88)	**0.001**
Platelets	1.18 (0.97–1.32)	0.216	1.02 (0.69–1.31)	0.424
D-dimers	3.82 (2.33–5.65)	<0.001	3.14 (2.06–4.38)	**<0.001**
Ferritin	1.70 (1.28–2.57)	<0.001	1.38 (1.01–1.84)	**0.004**
IL-6	5.23 (2.23–6.11)	<0.001	3.49 (2.28–4.70)	**<0.001**
SCL-90	2.14 (1.47–2.89)	<0.001	1.33 (1.15–2.01)	**0.033**

COPE—Coping Orientation to Problems Experienced Inventory; SCL-90—Symptom Checklist-90.

## Data Availability

Data available on request.
